# Genome sequence of the root-knot nematode *Meloidogyne luci*

**DOI:** 10.21307/jofnem-2020-025

**Published:** 2020-03-17

**Authors:** Nik Susič, Georgios D. Koutsovoulos, Cristian Riccio, Etienne G. J. Danchin, Mark L. Blaxter, David H. Lunt, Polona Strajnar, Saša Širca, Gregor Urek, Barbara Gerič Stare

**Affiliations:** 1 Agricultural Institute of Slovenia, Plant Protection Department, Ljubljana, Slovenia; 2 Institut Sophia Agrobiotech, INRA, Université Côte d’Azur, CNRS, Sophia-Antipolis, France; 3 Wellcome Sanger Institute, Wellcome Genome Campus, Hinxton, CB10 1SA, UK; 4 Institute of Evolutionary Biology, University of Edinburgh, Edinburgh, Scotland, EH9 3JT, UK; 5 University of Hull, Kingston upon Hull, UK

**Keywords:** Genome, Genomics, Illumina, *Meloidogyne luci*, PacBio, Root-knot nematode.

## Abstract

Root-knot nematodes from the genus *Meloidogyne* are polyphagous plant endoparasites and agricultural pests of global importance. Here, we report the high-quality genome sequence of *Meloidogyne luci* population SI-Smartno V13. The resulting genome assembly of *M. luci* SI-Smartno V13 consists of 327 contigs, with an N50 contig length of 1,711,905 bp and a total assembly length of 209.16 Mb.

Root-knot nematodes (RKN) from the genus *Meloidogyne* parasitize a wide range of host plants and have a global distribution. They are considered the most important group of plant-parasitic nematodes (Jones et al., 2013). Field infestations result in economic damage due to reduction or loss of crop yield with estimated global annual losses of $110bn (Danchin et al., 2013; Bebber et al., 2014). Among RKN, the tropical species belonging to *Meloidogyne* Clade I reproduce asexually by mitotic parthenogenesis (except *M. floridensis*) and parasitize a broader range of hosts than their sexual relatives (Castagnone-Sereno and Danchin, 2014). Several genomes of Clade I tropical *Meloidogyne* spp. have been sequenced (Abad et al., 2008; Lunt et al., 2014; Blanc-Mathieu et al., 2017; Szitenberg et al., 2017) and have revealed them to be complex allopolyploids with heterozygous duplicated genome regions and abundant transposable elements (Blanc-Mathieu et al., 2017; Szitenberg et al., 2017). Previous genome assemblies largely relied on short-read next-generation sequencing which limited the contiguity of the assemblies. Sato et al. (2018) found that applying long-read sequencing technologies such as Pacific Biosciences single-molecule real-time (SMRT) significantly improved the contiguity of their *Meloidogyne arenaria* assembly.

The species belonging to the *Meloidogyne ethiopica* group include the closely related species *M. ethiopica*, *M. inornata* and *M. luci* (Gerič Stare et al., 2019). The phylogenetic positions of different populations of *M. ethiopica* group species within Clade I *Meloidogyne* are incompletely resolved. Isolated specimens of *M. luci* in Europe were previously misidentified as *M. ethiopica* due to their high similarity (Gerič Stare et al., 2017). We used long-read Pacific Biosciences Sequel and short-read Illumina HiSeqX sequencing data to produce a high-quality *Meloidogyne luci* genome assembly. The *M. luci* population SI-Smartno was isolated from tomato plants grown in a commercial production greenhouse in Šmartno, Slovenia (Gerič Stare et al., 2018). A line (V13) was reared from the progeny of a single female and multiplied on tomato (*Solanum lycopersicum* “Val”). Nematode eggs were obtained by hypochlorite extraction (Hussey and Barker, 1973) and cleaned by sucrose flotation (McClure et al., 1973). Genomic DNA (gDNA) was obtained by phenol-chloroform extraction from the nematode eggs ground in liquid nitrogen. Following fluorometric quantification (Qubit; Thermo Fisher Scientific), a total of 6.64 μg of gDNA was used for Illumina whole-genome sequencing (WGS) on HiSeqX platform. 150 bp paired-end reads were generated from 350 bp insert TruSeq DNA PCR-Free libraries, yielding 206,071,630 reads (30.9 Gb). Reads were quality checked with FastQC v0.11.8 (Andrews, 2018) and trimmed with Trimmomatic v0.36 (Bolger et al., 2014) using the Phred quality score cutoff at 20. Prior to Pacific Biosciences SMRT sequencing on the Sequel, gDNA was assessed using the Femto Pulse system (Agilent) and a total of 10 μg of gDNA was used. The SMRTbell Express template prep kit (Pacific Biosciences) was used to prepare PacBio library >20 kb using standard protocol without shearing (Procedure & Checklist – Preparing >15 kb Libraries Using SMRTbell® Express Template Preparation Kit). Blue Pippin (Sage Science, MA, USA) was used for size selection (25 kb cutoff). Libraries were sequenced on the Sequel using v2.1 Sequencing and Binding kits generating 3,617,847 reads (42.4 Gb). We generated approximately 150-fold and 200-fold genome coverage using Illumina and PacBio data, respectively. Adapter and barcode sequences were filtered out within the Sequel instrument and assembled with HGAP4 pipeline (SMRT Link suite v5.1.0.26412, Pacific Biosciences) and polished with Pilon (Walker et al., 2014) using trimmed Illumina data.

The assembled *M. luci* SI-Smartno genome consists of 327 contigs with a minimum contig length of 10,147 bp and N50 contig length of 1,711,905 bp. The total length of assembly is 209.16 Mb. Smudgeplot v0.1.3 (Ranallo-Benavidez et al., in press) and Jellyfish v1.0 (Marçais and Kingsford, 2011) were used to estimate genome ploidy based on the counting of k-mers (k = 21) on short-read data. The genome is estimated to be triploid (AAB). Blobtools (Laetsch and Blaxter, 2017) was used to assess contaminant DNA presence (Fig. 1B). The assembly is currently the most contiguous RKN assembly (Table 1) available with an estimated coverage of 95.16% of the coding space based on Core Eukaryotic Genes Mapping Approach (CEGMA) analysis (Parra et al., 2007) and the average number of CEGs at 2.88 supports the triploid genome model (Fig. 1A). The polished assembly was 88.1% complete based on the eukaryote set (*n* = 303) of Benchmarking Universal Single-Copy Orthologs (Simão et al., 2015). The assembly of *M. luci* SI-Smartno can now be used to determine the correct phylogenetic position of the clade, identification of genetic changes related to the origins of virulence, and in the study of evolutionary history of this organism.

**Table 1.** Summary statistics of the *Meloidogyne luci* genome assembly compared to the genome assemblies of other *Meloidogyne* spp. currently available in DDBJ/ENA/GenBank.

Species	Strain/isolate desig-nation	Accession (DDBJ/ENA/GenBank)	Assembly size (Mb)	Genome coverage	Number of contigs/scaffolds	N50	GC content (%)	Number of pre-dicted genes	CEGMA score (% complete)	Reference
*M. luci*	SI-Smartno V13	ERS3574357	209.16	200	327	1,711,905	30.2	n/a	95.2	This study
*M. incognita*	Morelos	GCA_000180415.1	82.10	5	9,538	12,786	31.4	19,212	77	Abad et al. (2008)
*M. incognita*	W1	GCA_003693645.1	121.96	100	33,351	16,520	30.6	24,714	83	Szitenberg et al. (2017)
*M. incognita*	V3	GCA_900182535.1	183.53	100	12,091	38,588	29.8	45,351	97	Blanc-Mathieu et al. (2017)
*M. javanica*	VW4	GCA_003693625.1	150.35	300	34,316	14,128	30.2	26,917	90	Szitenberg et al. (2017)
*M. javanica*	–	GCA_900003945.1	235.80	100	31,341	10,388	29.9	98,578	96	Blanc-Mathieu et al. (2017)
*M. floridensis*	–	GCA_000751915.1	96.67	200	58,696	3,698	30.0	n/a	58.1	Lunt et al. (2014)
*M. floridensis*	SJF1	GCA_003693605.1	74.85	100	8,887	13,261	30.2	14,144	84	Szitenberg et al. (2017)
*M. arenaria*	HarA	GCA_003693565.1	163.75	100	46,436	10,504	30.3	30,308	91	Szitenberg et al. (2017)
*M. arenaria*	–	GCA_900003985.1	258.07	100	26,196	16,462	29.8	103,001	95	Blanc-Mathieu et al. (2017)
*M. arenaria*	A2-O	GCA_003133805.1	284.05	60	2,224	204,551	30.0	n/a	94.8	Sato et al. (2018)
*M. enterolobii*	L30	GCA_003693675.1	162.97	200	42,008	10,552	30.2	31,051	81	Szitenberg et al. (2017)
*M. graminicola*	IARI	GCA_002778205.1	38.19	180	4,304	20,482	23.1	10,196	84.3	Somvanshi et al. (2018)
*M. hapla*	VW9	GCA_000172435.1	53.01	10	3,450	37,608	27.4	14,420	94.8	Opperman et al. (2008)

Note: n/a, not assessed.

**Figure 1:**
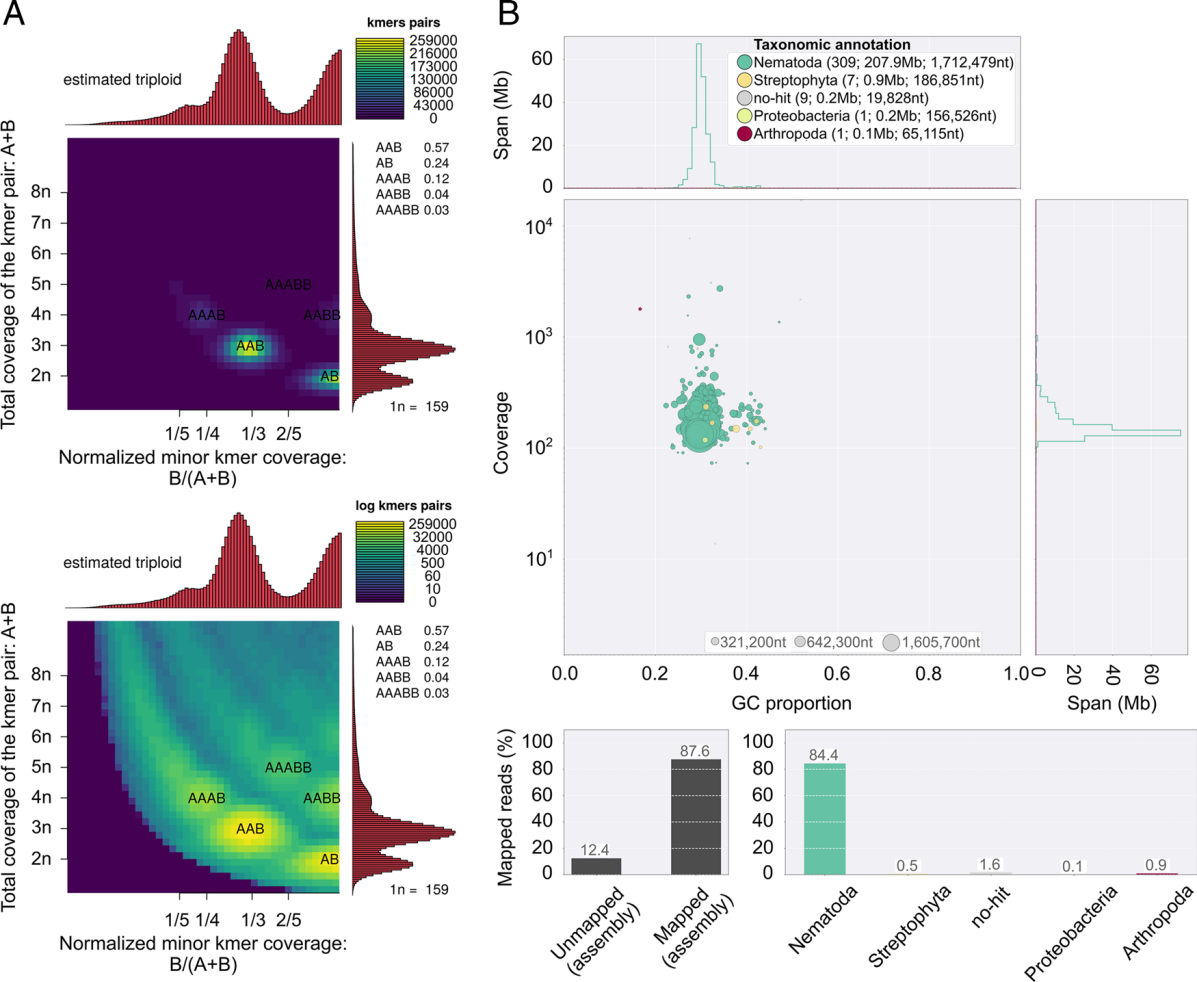
Genome ploidy estimation and contaminant analysis of the *Meloidogyne luci* SI-Smartno genome assembly. (A) Smudgeplots showing the coverage and distribution of k-mer pairs that fit to triploid genome model. (B) Blobplot showing the lack of contamination of assembly by foreign (non-Nematoda) genetic material.

## Data availability and accession number(s)

Procedural information concerning the genome assembly and analysis presented in this paper can be found at the GitHub repository at https://github.com/CristianRiccio/mluci. The sequences have been deposited in DDBJ/ENA/GenBank under the accession number ERS3574357.
